# Supplementary material for: Mode-locked short pulses from an 8 μm wavelength semiconductor laser
Source: Nat Commun. 2020 Nov 13;11:5788. doi: 10.1038/s41467-020-19592-1 (PMC7666187; doi:10.1038/s41467-020-19592-1)
Supplement: Supplementary file 1 — Supplementary Information [file 41467_2020_19592_MOESM1_ESM.pdf]

Supplementary material
Mode-locked short pulses from an 8 µm wavelength semiconductor laser

Johannes Hillbrand,[1,2,*] Nikola Opačak,[1] Marco Piccardo,[2,3] Harald Schneider,[4] Gottfried Strasser,[1] Federico Capasso,[2] and Benedikt Schwarz[1,2,†]

[1]*Institute of Solid State Electronics, TU Wien, Gusshausstrasse 25-25a, 1040 Vienna, Austria*
[2]*John A. Paulson School of Engineering and Applied Sciences, Harvard University, Cambridge, USA*
[3]*CNST – Fondazione Istituto Italiano di Tecnologia, Via Pascoli 70/3, 20133 Milano, Italy*
[4]*Institute of Ion Beam Physics and Materials Research, Helmholtz-Zentrum Dresden, Germany*

SUPPLEMENTARY NOTE 1: THEORETICAL MODEL

All of the numerical simulations that are conducted in this work utilize the theoretical formalism developed recently in [1]. In the mentioned work, authors analyze frequency-modulated (FM) comb formation in QCLs. In our study, the formation of amplitude-modulated (AM) combs is analyzed, however, the same formalism can be applied. The benefit of doing so is twofold due to the sheer simplicity of the equation system and highly efficient numerical implementation.

The formalism developed in [1] will be briefly described here. The theoretical background of the approach in the mentioned work is based on the well-known Maxwell-Bloch equations (MBE). MBE define a system of coupled differential equations for the complex electric field envelope in the laser cavity, macroscopic polarization and carrier population inversion. The authors use this system of equations as a starting point and utilize the fact that fast gain media (such as QCLs) possess short carrier lifetimes, which are in the order of picoseconds. This allows elimination of the differential equation for the population inversion via Taylor expansion. The differential equation for the macroscopic polarization is eliminated in the same way. Thus, the whole Maxwell-Bloch system of equations is replaced with a single master equation for the complex field envelope:

$$\begin{aligned}\left(\frac{n}{c}\partial_t \pm \partial_z\right)E_\pm &= \frac{g(P)}{2}\Big[E_\pm - T_2\partial_t E_\pm + T_2^2\partial_t^2 E_\pm\Big] \\ &- \frac{g(P)T_g}{2T_1P_{\mathrm{sat}}}\Big[|E_\mp|^2E_\pm - (T_2+T_{\mathrm{g}})|E_\mp|^2\partial_t E_\pm - (T_2+T_{\mathrm{g}})E_\pm E_\mp\partial_t E_\mp^* - T_2E_\pm E_\mp^*\partial_t E_\mp\Big] \\ &+ i\frac{k''}{2}\partial_t^2 E_\pm + i\beta\left(|E_+|^2+|E_-|^2\right)E_\pm - \frac{\alpha_w}{2}E_\pm.\end{aligned} \tag{1}$$

Here $E_\pm$ are the complex amplitudes of the two counterpropagating field envelopes in the laser cavity, n is the refractive index, c the speed of light, T_1 the carrier non-radiative lifetime, T_2 the dephasing time, $T_g = (T_1^{-1}+4k^2D)^{-1}$ is the carrier "grating" lifetime, where k and D stand for the wavenumber and diffusion coefficient. We have introduced furthermore k'' as the group velocity dispersion coefficient (GVD) and β the Kerr nonlinearity. Saturated gain is given with $g(P) = g_0/(1+P/P_{\mathrm{sat}})$, where $P_{\mathrm{sat}} = E_{sat}^2 = (2\hbar^2)/(\mu^2T_1T_2)$ is the saturation power and $P = |E_+|^2+|E_-|^2$ is the normalized power, μ is the dipole moment. The power loss coefficient is α_w and the unsaturated gain is $g_0 = (\Gamma\mu^2\omega_0T_1T_2J)/(\hbar n\varepsilon_0 cL)$, with Γ the confinement factor, J the pumping current normalized to the electron charge and multiplied with the sheet density and L the QCL active region period length.

The simulated cavity consists of two parts - a long section where the pumping current is constant and a short section next to one facet where the pumping current is modulated. The length of the short section is taken to be 10% of the total cavity length. The current in the modulation section is modeled with a simple sinusoidal modulation:

$$J = J_{DC} + J_{mod}\sin(\omega_{mod}t), \tag{2}$$

where ω_{mod} is the modulation frequency, J_{DC} represents the DC bias and the amplitude of the modulation depth is given with J_{mod}. The transparency current could be added directly. The small deviations of the the pulse widths and shapes between simulation and experiment can be attributed the linear dependence between the current and the sinusoidal modulation in equation (2). A more complex model that uses a bias dependent tunneling rate, similarly to Ref. [2] could further improve the quantitative agreement of the pulse shape and width.

The values of the used parameters are:

Symbol	Description	Value
T_1	Carrier lifetime	1 ps
T_2	Dephasing time	60 fs
n	Refractive index	3.3
D	Diffusion coefficient	46 cm^2/s
α_w	Waveguide power losses	4 cm^{-1}
μ	Dipole matrix element	2 nm $\times$ e
n_{tot}	Sheet density	6×10^{10} cm^{-2}
R_l, R_r	Terminal facets reflectivity	0.3
Γ	Confinement factor	0.6
L	Doping thickness	580 Å
L_c	Cavity length	4 mm
λ_0	Central wavelength	8 μm

Supplementary Table I – parameters used for the numerical simulation.

SUPPLEMENTARY NOTE 2: SYNCHRONIZATION STATES IN MODE-LOCKED QUANTUM CASCADE LASERS

The concept of in-phase and anti-phase synchronization in mode-locked QCLs is introduced in Fig. 2 of the main paper. This section contains further detailed experimental and theoretical results about this phenomenon.

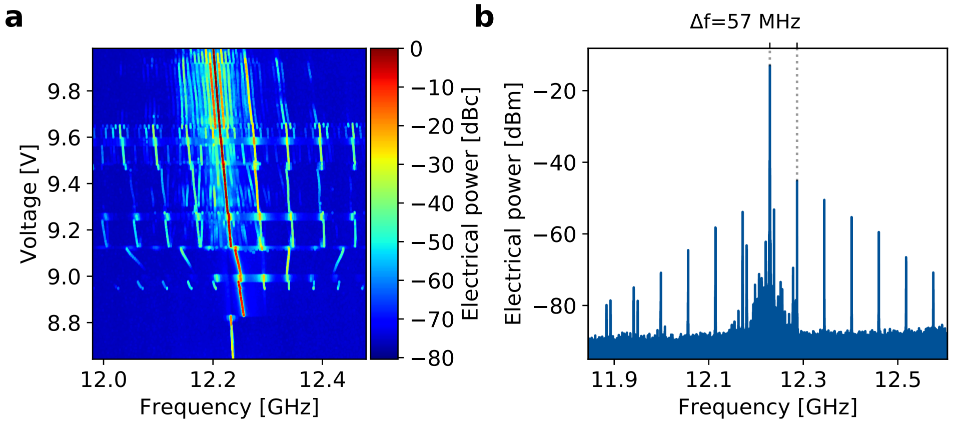

Supplementary Figure 1 – **a**: RF spectrum around the beatnote f^0_{rep} of the free-running QCL depending on the bias. Extracting the RF beating directly from the optimized modulation section enables to measure the beatnote with a signal to noise ratio over 80 dB, unveiling several weaker features in the vicinity of the main beatnote at f^0_{rep}. **b**: RF spectrum at 9.35 V bias, corresponding to the driving conditions in Figs. 1e,f of the main text. Apart from the strong peak at f^0_{rep}, several weaker equidistant sidepeaks separated by 57 MHz are clearly visible. Strikingly, the main peak and its strongest sidepeak are located at the two synchronization frequencies observed in Figs. 1e,f of the main text. These frequencies correspond to the beatnote frequency of the free running QCL comb and the actively mode-locked QCL comb. This suggests that the two synchronization states are already evident in the beatnote of the free-running laser, which provides further reinforcement for the analogy to the system of coupled oscillators..

The simulated and measured evolution of the QCL spectrum as the modulation frequency f_{mod} is swept across the synchronization frequency f_0 is shown in supp. Fig. 2. In the numerical studies , the dependence on the Kerr non-linearity β is analyzed additionally ($\beta < 0$ in supp. Fig. 2a, $\beta = 0$ in supp. Fig. 2b and $\beta > 0$ in supp. Fig. 2c). It is seen that for f_{mod} bein sufficiently far from the synchronization frequency f_0, the spectrum consists of a single Gaussian shaped lobe. However, an interesting phenomenon occurs for nonzero Kerr non-linearity present in the laser cavity. As f_{mod} approaches f_0, the spectrum shifts and becomes considerably broader, developing several weaker side lobes. The spectral shift and and frequency of the sidelobes is antisymetric for opposite signs of the Kerr non-linearity. For $\beta = 0$, this is not the case and the spectrum does not change as function of f_{mod} (supp. Fig. 2b). The evolution of the measured spectra as the f_{mod} is swept across the anti-phase synchronization frequency (supp. Fig. 2d) and the in-phase synchronization freqeuncy (supp. Fig. 2e). Strikingly similar behavior as in supp. Fig. 2a is observed, indicating that the value of the Kerr non-linearity in the investigated devices is negative. This conclusion also matches the findings in [1].

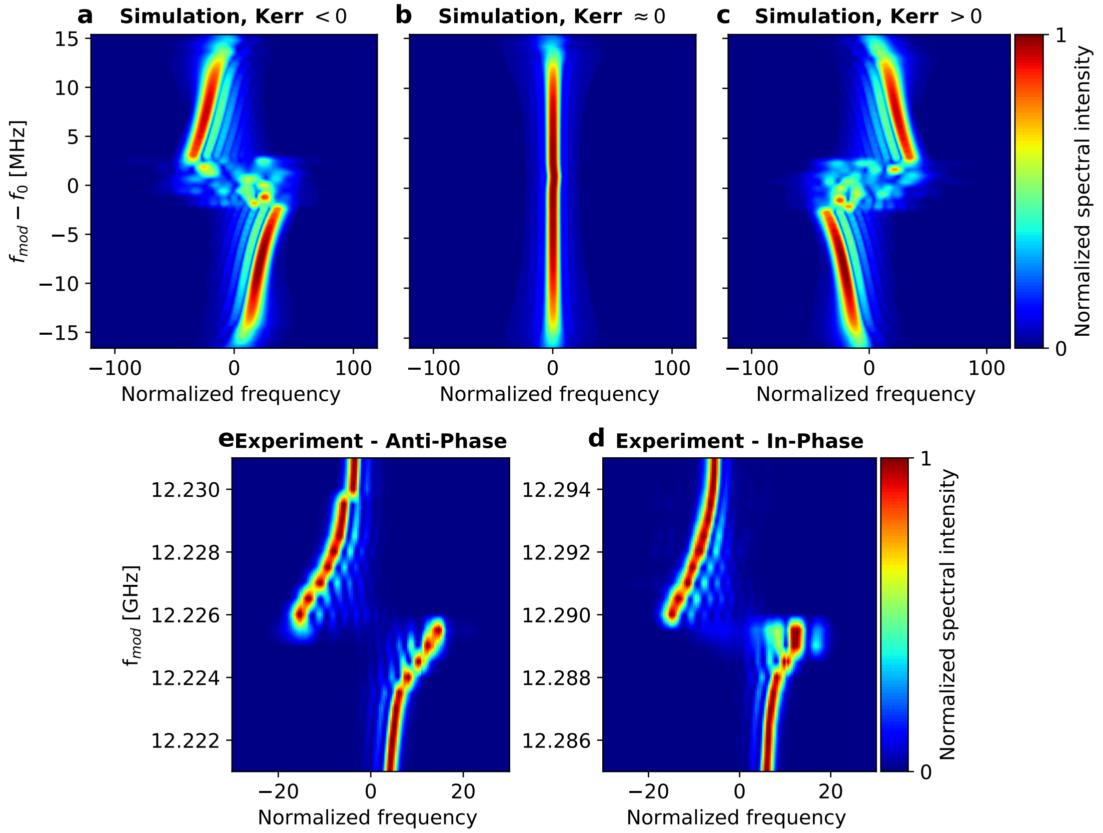

Supplementary Figure 2 – Results of the simulated QCL spectrum using the Master equation 1 as the modulation frequency f_{mod} is swept across a synchronization frequency f_0, assuming: **a** negative Kerr non-linearity **b** zero Kerr non-linearity and **c** postiive Kerr non-linearity. **d**: Evolution of the measured QCL spectrum as f_{mod} is swept across the anti-phase synchronization frequency f^0_{rep} at 12.226 GHz. **e**: Evolution of the measured QCL spectrum as f_{mod} is swept across the in-phase synchronization frequency at 12.290 GHz. The optical frequency of the spectra is normalized to the roundtrip frequency.

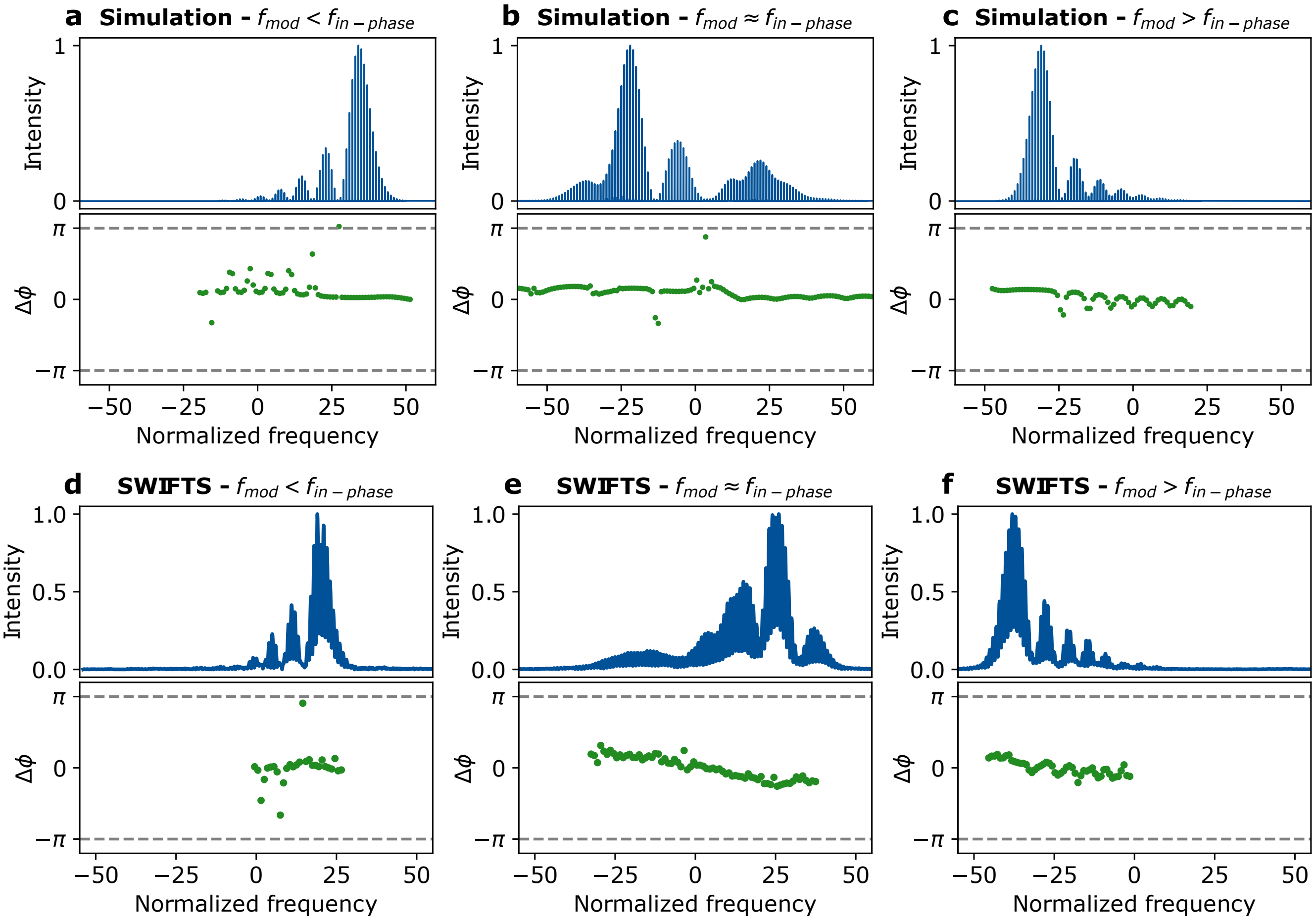

Supplementary Figure 3 – Detailed view of the simulated spectra shown in supp. Fig. 2 and intermode difference phases $\Delta\phi$ when modulating **a**: below the in-phase synchronization frequency $f_{\mathrm{in-phase}}$ **b** close to $f_{\mathrm{in-phase}}$ and **c** above $f_{\mathrm{in-phase}}$. Both the shift of the spectrum and the sidelobes can be clearly observed. The measured spectra at these modulation frequencies are shown in **d, e** and **f**. The comparison between the simulated and measured spectra shows excellent agreement.

SUPPLEMENTARY NOTE 3: WAVEFORM RECONSTRUCTION FROM IAC MEASUREMENTS

As discussed in the main text, the interferometric autocorrelation (IAC) measurements obtained using a two-photon quantum-well infrared photodetector (2-QWIP) provide a clear proof for the generation of mode-locked pulses in our lasers based on the well-known 8 : 1 peak-to-background ratio of the IAC trace [3]. In this section we will show that more than this basic information can be retrieved from the IAC data, demonstrating that also the temporal waveform can be reconstructed. The following results further strengthen the agreement with the SWIFTS measurements.

Naganuma *et al.* introduced in Ref. [4] an iterative algorithm that allows to reconstruct the temporal waveform of a pulsed laser by using as an input only the second-order IAC trace and the spectrum of the laser. More recently, this algorithm was expanded and proven to be successful also for the reconstruction of frequency-modulated lasers, whose temporal waveforms are strikingly different from those of mode-locked lasers due to the absence of amplitude modulations [5]. Thus this algorithm can be considered to be generally valid for the reconstruction of an arbitrary waveform. Here we employ it to obtain the waveform of the mode-locked lasers presented in this work.

Description of the iterative algorithm

The second-order IAC trace encompasses the intensity and quadratic field autocorrelation traces. Such signals are superimposed in the interferogram in the time domain but can easily be separated by Fourier analysis as they have different carrier frequencies. The intensity autocorrelation is slowly varying and its Fourier transform, $|\hat{I}(f)|$, lies close to zero frequency, while the quadratic spectrum, $|\hat{u}(f)|$, is found at the second harmonic of the optical carrier of the laser [4].

A flowchart of the algorithm is shown in Fig. 4. The initial inputs are the measured fundamental spectrum of the laser (obtained from the IAC trace measured with a linear detector) and arbitrarily picked spectral phases, which constitute an initial guess. Based on this input we then compute the trial time-domain electric field, intensity and quadratic field functions to initialize the loop. At every iteration, in the Fourier space the moduli of the two functions are replaced by the experimental spectra while the corresponding spectral phases are kept, thus automatically refining the trial functions. The algorithm terminates when the three residuals computed using the trial moduli and the experimental spectra simultaneously become sufficiently small.

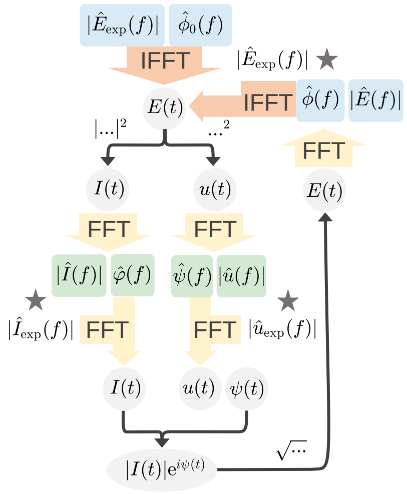

Supplementary Figure 4 – Diagram showing the iteration scheme of the algorithm which takes as inputs an initial guess $\hat{\phi}_0(f)$ for the spectral phase of the electric field together with the spectral amplitudes of $|\hat{E}(f)|$, $|\hat{I}(f)|$ and $|\hat{u}(f)|$, and gives as outputs the temporal waveform of the electric field $E(t)$ together with its phase $\phi(t)$. The residuals of the different spectral functions are computed at different check-points in each iteration marked by stars in the diagram.

Result of the reconstruction

The temporal waveform obtained from the experimental linear and second-order IAC traces (Fig. 5a) using the iterative algorithm is shown in Fig. 5b. The waveform shows the presence of pronounced peaks of intensity with a full-width half-maximum of approximately 8 ps. We have verified that the reconstruction is independent from the nature of the initial guess of the spectral phases used at the input of the iterative algorithm ($\hat{\phi}_0(f)$ in Fig. 4), which was varied between a random guess, a parabolic phase profile and a flat phase profile, giving essentially the same result. The robustness of the reconstruction was also indicated by the fast convergence of the algorithm and small residuals. The reconstructed waveform can be compared with the one obtained using the SWIFTS technique for the same laser (Fig. 5b). A very good agreement is observed between the two results. We attribute the larger noise of the IAC waveform to the iterative nature of the reconstruction technique (as opposed to the direct reconstruction of SWIFTS), and to the lower signal-to-noise ratio of the 2-QWIP measurement (as compared to the signal of the fast linear detector used in SWIFTS). These results corroborates the validity of the two waveforms reconstruction techniques and provide an additional evidence for the pulse generation in our mode-locked QCLs.

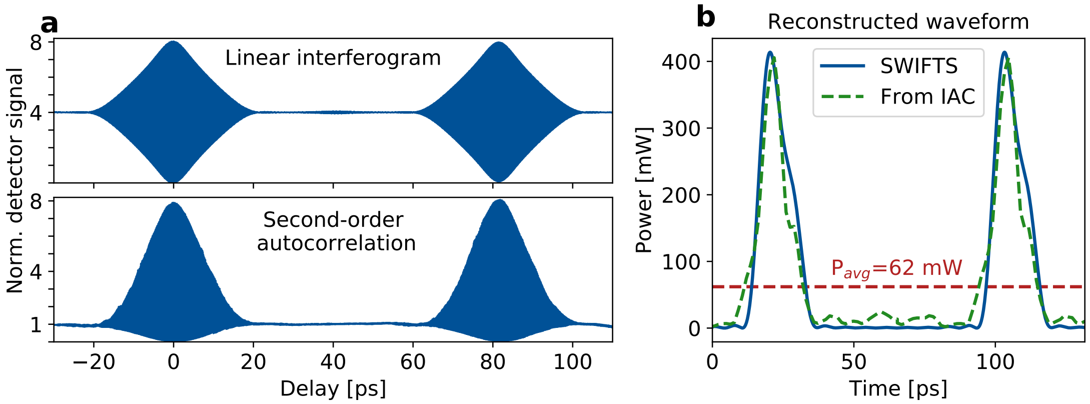

Supplementary Figure 5 – **a**: Experimental autocorrelation traces measured using a linear (top) and a quadratic (bottom) photodetector. The source is a mode-locked QCL. **b**: Comparison of the waveforms reconstructed for the same mode-locked QCL using the SWIFTS technique and the iterative algorithm based on the second-order IAC trace.

SUPPLEMENTARY NOTE 4: INFLUENCE OF THE PUMPING CURRENT ON THE PULSE WIDTH

The gain of QCLs is saturated by intense pulses circulating in the cavity. As a consequence, a pulse experiences more gain at its wings than at its intensity peak, which can lead to pulse broadening. Both the SWIFTS data and interferometric autocorrelations presented in Fig. 2 of the main text suggest that the pulse width is growing when the driving current is increased. The width of a pulse is mainly determined by the width of the spectrum and the phase relationship of the modes. If the latter are synchronized perfectly in-phase, the duration of the pulse is proportional to the inverse of the spectral width and said to be transform limited. If the intermode difference phases are not constant, the pulse becomes chirped and its duration grows as well. Supplementary Fig. 6 shows the simulated (left) and measured (right) pulses emitted by the QCL for increasing pumping current. Both the simulations and the experimental results show that the phases remain almost perfectly synchronized in-phase for all driving current values, resulting in transform-limited pulses up to rollover. However, the spectral width decreases for increasing driving current. As a consequence, the pulse width increases by roughly 60 % from threshold to rollover.

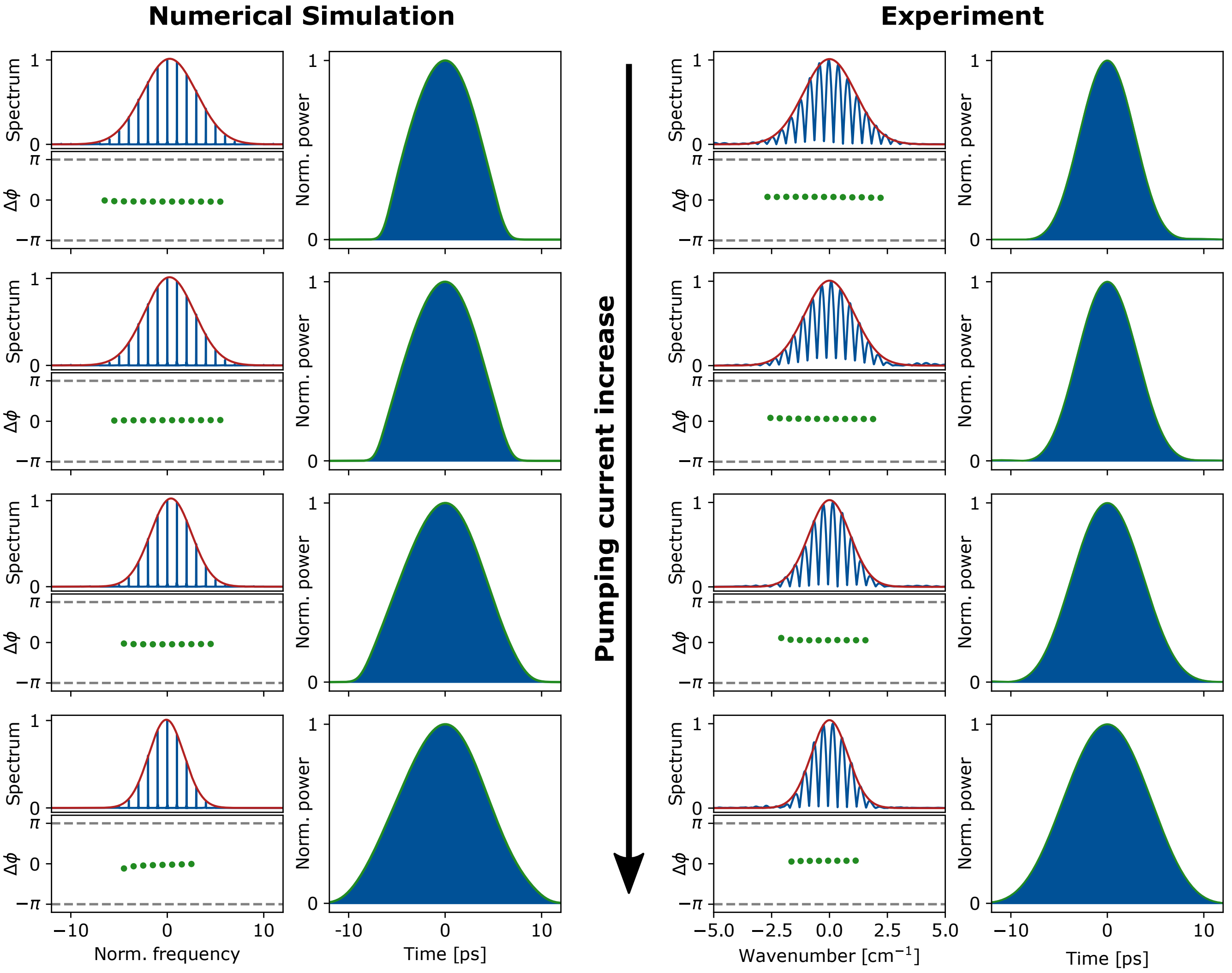

Supplementary Figure 6 – Simulation (left) and SWIFTS characterization (right) of the mode-locked QCLs for increasing pumping current (top to bottom).

SUPPLEMENTARY NOTE 5: INFLUENCE OF DISPERSION

Free-running QCL frequency combs, which are based on the gain non-linearity acting as a locking mechanism and do not require external modulation, are strongly affected by dispersion[6–8]. Excessive dispersion is known to cause a high phase-noise regime, where the linewidth of the QCL is strongly increased. The increased phase-noise in this regime is detrimental for applications such as dual-comb spectroscopy and requires computational algorithms to be corrected. In contrast, the QCL frequency combs presented in this work are based on strong external modulation to lock the modes. Fig. 7 illustrates the influence of dispersion on the pulse dynamics using the numerical model described above. At zero group velocity dispersion (GVD), the intermode difference phases $\Delta\phi$ synchronize in-phase with negligible chirp resulting in a transform-limited pulse width of 12.5 ps. At 1500 fs^2/cm and 3000 fs^2/cm GVD, $\Delta\phi$ acquire a slight linear chirp, but the pulse width increases by less than 0.5 ps. $\Delta\phi$ is directly related to the spectral group delay with 2π corresponding to one cavity roundtrip period $\tau_{rt} = 81$ ps. Hence, the phase range occupied by $\Delta\phi$ at 3000 fs^2/cm of 0.15 rad results in a difference in group delay of less than 2 ps, which considerably smaller than the transform-limited pulse width. As a result, we conclude that the pulse width at this stage is limited by the spectral width, and not dispersion.

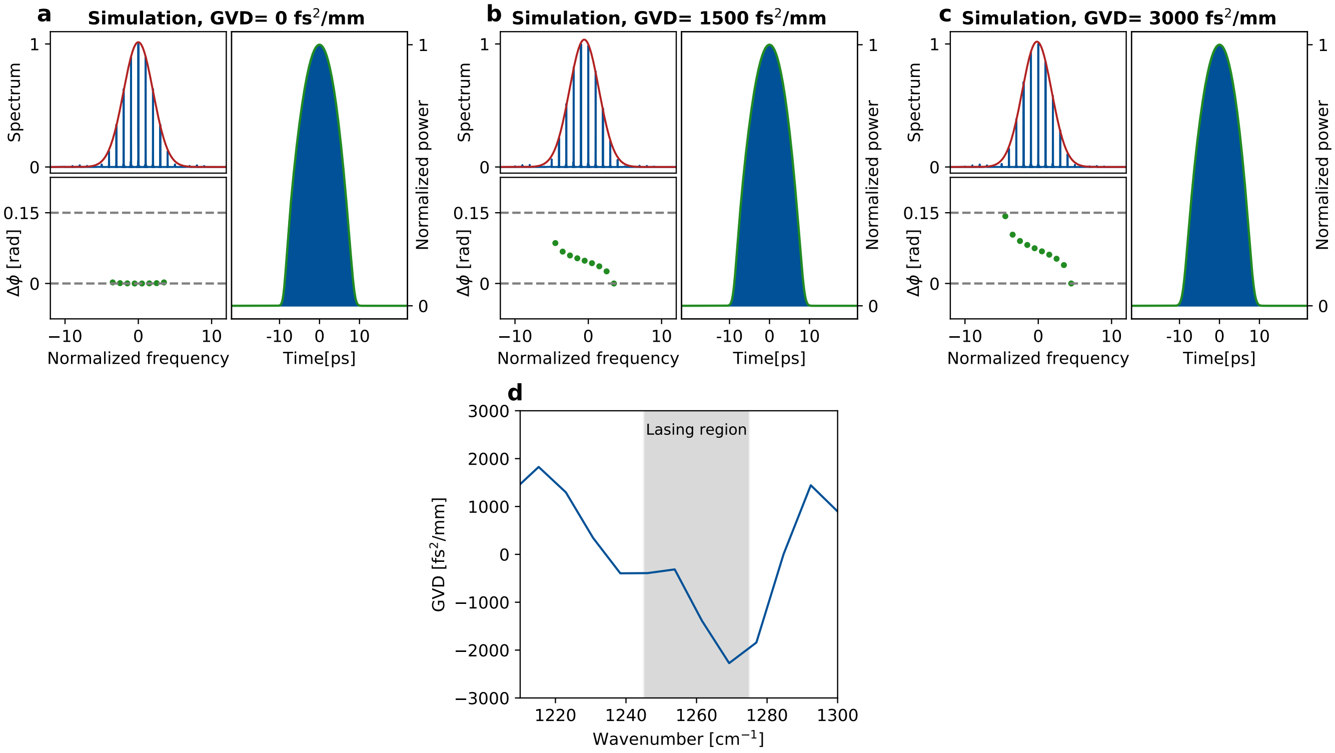

Supplementary Figure 7 – Simulation of the influence of group velocity dispersion on the formation of short pulses in QCLs for **a**: 0 fs^2/cm, **b**: 1500 fs^2/cm and **c**: 3000 fs^2/cm. **d**: Dispersion of the investigated devices measured using the Fourier transform method[9].

SUPPLEMENTARY NOTE 6: FREQUENCY RESPONSE OF THE MODULATION SECTION

Ensuring a high-speed frequency response of the modulation section is essential to achieve the large modulation depth required for active mode-locking. There are at least two parameters,which can influence the modulation capabilities of semiconductor lasers: the speed, up to which the gain medium can be modulated due to its internal carrier dynamics, and the capacitance of the laser. Due to fast intersubband scattering on the sub-picosecond timescale, QCLs can be modulated efficiently up to tens of GHz and do not show relaxation oscillations [10], in contrast to many interband lasers. Hence, the limiting property of the modulation response of QCLs is in most cases their parasitic capacitance.

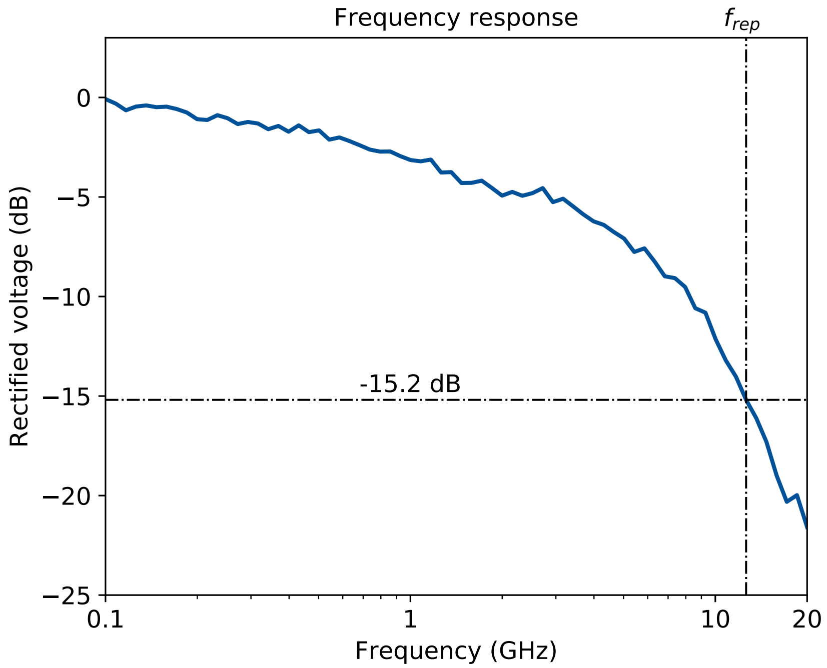

Supplementary Figure 8 – Frequency response of a 300 µm short modulation section measured using microwave rectification.

We have measured the frequency response of a 300 µm short modulation section using a microwave rectification technique (supp. Fig. 8). At the cavity roundtrip frequency of a 3.5 mm long device of approximately 12.3 GHz, the modulation setup (including coaxial cables and RF tips used to inject the signal as well as the parasitic capacitance of the QCL) shows a loss of roughly 15 dB. Hence, around 160 mW of RF power are injected into the laser when the power of the modulation source is 5 W. This could be improved considerably by further minimizing the parasitic capacitance of the modulation section and using longer devices with a smaller cavity roundtrip frequency.

SUPPLEMENTARY NOTE 7: QCL STRUCTURE

The bi-functional QCLs were grown using lattice matched InGaAs/InAlAs on a n^{+}-InP substrate in a metalorganic vapour-phase epitaxy (MOVPE) system. The layer structure including waveguide claddings, active region and contact layers is given below. Furthermore, chirped superlattices were inserted between the contact layers and the low-doped InP claddings to reduce the series resistance. The exact structure can be found in Ref. [11].

Thickness (nm)	Material	Doping (cm^{-3})	Name
350 µm	n^{+}-InP	$2.5 \cdot 10^{18}$	substrate
3500	n-InP	$3 \cdot 10^{16}$	bottom cladding
			35× active region
5.5	InGaAs	-	-
2.1	InAlAs	-	-
6.7	InGaAs	-	-
1.1	InAlAs	-	-
1.9	InGaAs	-	-
3.9	InAlAs	-	-
2.9	InGaAs	-	-
2.3	InAlAs	-	-
2.95	InGaAs	-	-
2.4	InAlAs	-	-
3.3	InGaAs	-	-
2.0	InAlAs	-	-
3.6	InGaAs	$2 \cdot 10^{17}$	-
1.9	InAlAs	$2 \cdot 10^{17}$	-
3.8	InGaAs	$2 \cdot 10^{17}$	-
1.8	InAlAs	$2 \cdot 10^{17}$	-
4.0	InAlAs	$2 \cdot 10^{17}$	-
1.7	InAlAs	$2 \cdot 10^{17}$	-
4.2	InGaAs	-	-
1.7	InAlAs	-	-
4.6	InGaAs	-	-
2.2	InAlAs	-	-
3500	n-InP	$3 \cdot 10^{16}$	top cladding
700	n^{+}-InP	$7 \cdot 10^{18}$	-
200	n^{+}-InP	$1 \cdot 10^{19}$	-
200	InGaAs	$2.5 \cdot 10^{19}$	top contact

Supplementary Table II – QCL growth sheet.

SUPPLEMENTARY NOTE 8: COMPARISON OF FREE-RUNNING AND ACTIVELY MODE-LOCKED QCL FREQUENCY COMB

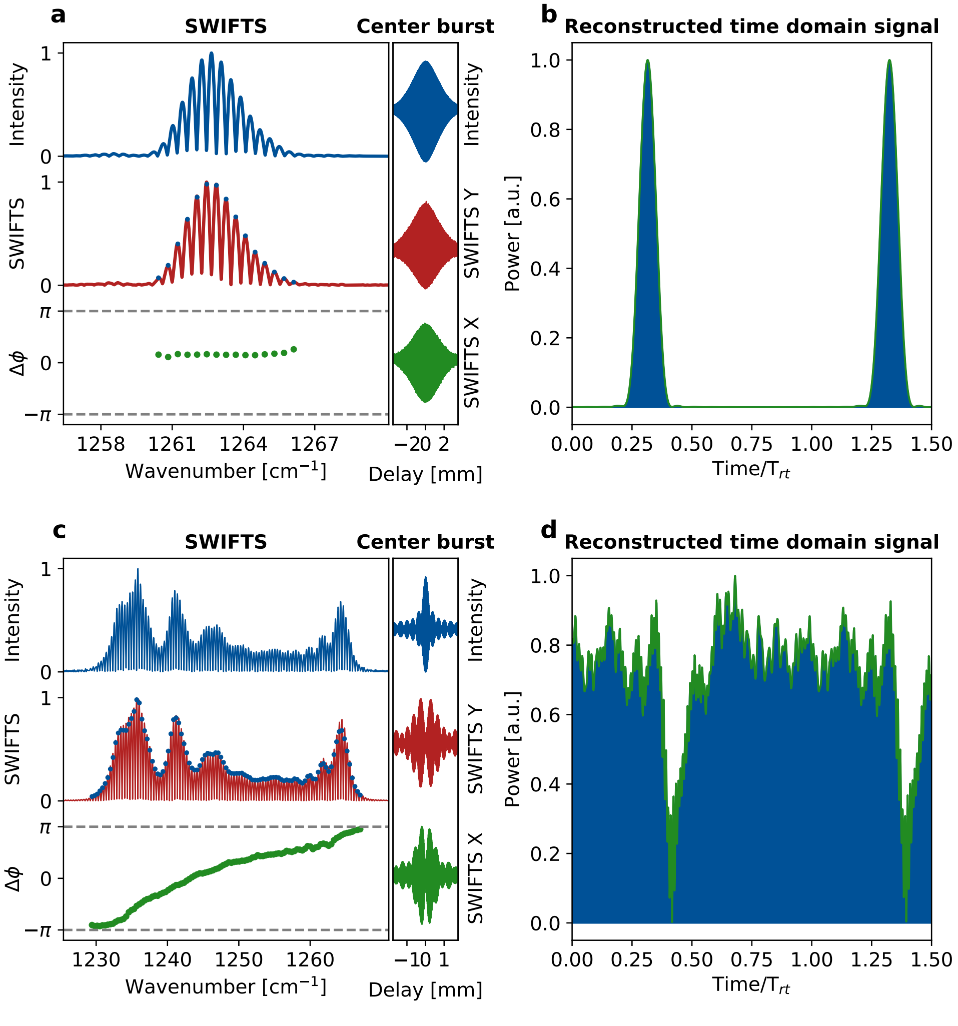

Supplementary Figure 9 – Comparison of the SWIFTS characterization and reconstructed waveform of the free-running and actively mode-locked QCL frequency comb. **a**: the spectrum of the actively mode-locked QCL consists of a single Gaussian-shaped lobe. The intermodal difference phases are synchronized in-phase. **b**: reconstructed waveform of the actively mode-locked QCL, showing a train of short pulses.**c**: the spectrum of the free-running QCL consists of several lobes. In contrast to **a**, the intermodal difference phases are linearly splayed over the full range of 2π. **d**: reconstructed waveform emitted by the free-running QCL frequency comb showing almost no amplitude modulation.

SUPPLEMENTARY NOTE 8: RF CROSSTALK IN THE INTEGRATED DETECTOR CONFIGURATION

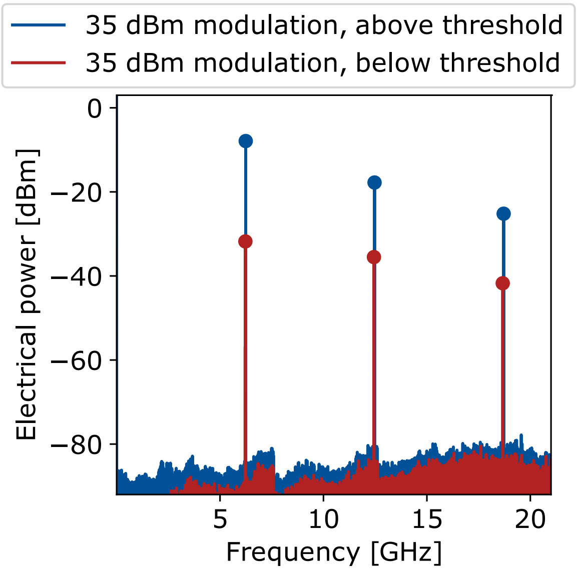

Supplementary Figure 10 – Comparison of the beatnote extracted from the integrated detector section when the QCL is below threshold (red curve), which corresponds to RF crosstalk, and when it the QCL is lasing. It can be seen that the amplitude of the extracted laser beatnote is more than 20 dB larger than the RF crosstalk amplitude.

SUPPLEMENTARY REFERENCES

* johannes.hillbrand@tuwien.ac.at
† benedikt.schwarz@tuwien.ac.at

[1] Opačak, N. & Schwarz, B. Theory of Frequency-Modulated Combs in Lasers with Spatial Hole Burning, Dispersion, and Kerr Nonlinearity. *Physical Review Letters* **123** (2019).

[2] Wang, Y. & Belyanin, A. Active mode-locking of mid-infrared quantum cascade lasers with short gain recovery time. *Optics Express* **23**, 4173 (2015).

[3] Wang, C. Y. *et al.* Mode-locked pulses from mid-infrared quantum cascade lasers. *Optics Express* **17**, 12929 (2009). URL `https://doi.org/10.1364/oe.17.012929`.

[4] Naganuma, K., Mogi, K. & Yamada, H. General method for ultrashort light pulse chirp measurement. *IEEE Journal of Quantum Electronics* **25**, 1225–1233 (1989).

[5] Piccardo, M. *et al.* Frequency-modulated combs obey a variational principle. *Phys. Rev. Lett.* **122**, 253901 (2019).

[6] Hugi, A., Villares, G., Blaser, S., Liu, H. C. & Faist, J. Mid-infrared frequency comb based on a quantum cascade laser. *Nature* **492**, 229–233 (2012).

[7] Johannes Hillbrand and Pierre Jouy and Mattias Beck and Jérôme Faist. Tunable dispersion compensation of quantum cascade laser frequency combs. *Optics Letters* **43**, 1746 (2018).

[8] Gustavo Villares and Sabine Riedi and Johanna Wolf and Dmitry Kazakov and Martin J. Süess and Pierre Jouy and Mattias Beck and Jérôme Faist. Dispersion engineering of quantum cascade laser frequency combs. *Optica* **3**, 252 (2016).

[9] Hofstetter, D. & Faist, J. Measurement of semiconductor laser gain and dispersion curves utilizing fourier transforms of the emission spectra. *IEEE Photonics Technology Letters* **11**, 1372–1374 (1999).

[10] Martini, R. *et al.* Absence of relaxation oscillation in quantum cascade lasers verified by high-frequency modulation. In *Technical Digest. Summaries of papers presented at the Conference on Lasers and Electro-Optics. Postconference Technical Digest (IEEE Cat. No.01CH37170)* (IEEE, 2001).

[11] Schwarz, B. *et al.* Watt-Level Continuous-Wave Emission from a Bifunctional Quantum Cascade Laser/Detector. *ACS Photonics* **4**, 1225–1231 (2017).
